# Problematic social networking site usage and substance use by young adolescents

**DOI:** 10.1186/s12887-018-1316-3

**Published:** 2018-11-23

**Authors:** Alessandra Buja, Luigi Gallimberti, Sonia Chindamo, Camilla Lion, Alberto Terraneo, Michele Rivera, Elena Marini, Luis Javier Gomez-Perez, Emanuele Scafato, Vincenzo Baldo

**Affiliations:** 1 Department of Cardiologic, Vascular, Thoracic Sciences and Public Health, University of Padova, Via Loredan, 18, 35131 Padova, Italy; 2 Novella Fronda Foundation for studies and applied clinical research in the field of addiction medicine, Padua, Italy; 3 2nd School of Hygiene and Preventive Medicine, University of Padova, Padova, Italy; 4 Department of Surgery, Oncology and Gastroenterology, University of Padova, Padova, Italy; 5 Department of Molecular Medicine; Laboratory of Public Health and Population Studies, Institute of Hygiene, University of Padova, Padova, Italy; 6 Direttore, WHO Collaborating Centre Research & Health Promotion on Alcohol and Alcohol-Related Health Problems, Rome, Italy; 7 Centro Nazionale Dipendenze e Doping-National Centre on Addictions and Doping, Rome, Italy; 8 Direttore, Osservatorio Nazionale Alcol - Director, National Observatory on Alcohol, Rome, Italy; 9 Hygiene and Public Health Unit, University of Padova, Padova, Italy

**Keywords:** Substance use, Adolescents, Problematic social networking site usage, Alcohol consumption, Energy drink consumption, Smoking

## Abstract

**Background:**

Substance use and abuse by young adolescents has become a serious issue for public health services, and several socio-environmental factors can influence how vulnerable a young adolescent may be to their appeal. The present study was devised to examine whether substance use in early adolescence is associated with problematic social networking site usage (PSNSU).

**Methods:**

In the academic year 2013–2014, secondary schools in Padua (north-eastern Italy) were involved in a survey called “Pinocchio”. A sample of 1325 pupils attending years 6 to 8 (i.e. aged from 11 to 13 years) completed self-administered questionnaires, in which PSNSU was measured by applying the DSM-IV criteria of dependence to identify any social network addiction disorder and its fallout on daily life. Multivariate analysis (ordered logistic regression) was performed to assess an adjusted association between young adolescents’ substance use and PSNSU.

**Results:**

The percentage of pupils classified as problematic social networking site users rose with age (from 14.6% in year 6 to 24.3% in year 7, and 37.2% in year 8), and it was higher in girls (27.1%) than in boys (23.6%). In a fully-adjusted model, PSNSU conferred a higher likelihood of being substance users (OR 2.93 95% CI 1.77–4.85).

**Conclusion:**

This study identified an association between PSNSU and the likelihood of substance use (smoking, alcohol and energy drink consumption), providing further evidence of the need to pay more attention to PSNSU in early adolescence.

## Background

Substance use and abuse by young adolescents is seen by public health services as a real cause for concern. Some of the socio-environmental factors that typically make children and young adolescents more or less vulnerable to substance abuse - such as peer pressure, and school and/or family environments - have already been thoroughly examined. There are other factors, however, that may have the potential to induce substance abuse [35], such as modern information and communication technologies, and particularly one that is very popular among adolescents, i.e. social networking sites (SNSs). In America, 76% of all people aged 13 to 17 use social media. Facebook is the dominant platform, with 71% of all adolescents using it. Instagram and Snapchat have also become increasingly popular, with 52% of teens using Instagram, and 41% using Snapchat. One in three American adolescents use Twitter and another one in three use Google Plus.

SNS usage provides new opportunities for exposure to unhealthy substances [36] because they are advertised more and more often on digital media, even among adolescents [45]. The content that adolescents report viewing on SNSs usually concerns pictures and comments posted by their friends [39], and researchers have found that as many as 25–37% of older teenagers post details about their alcohol drinking [38]. The content of such posts may give adolescents the impression that substance use as a normative behavior among peers of the same age and older. Compared with those who see alcohol use portrayed less frequently, adolescents who gain the impression from their elder peers’ Facebook profiles that it is normal to drink alcohol are at higher risk of developing an attitude shown to predict alcohol use [33]. SNS usage has become ever more popular and common, even to the point of giving rise to a clinical disorder associated with abuse-like signs, such as an excessive, compulsive online social networking. Several authors have claimed in recent times that this becomes a sort of behavioral addiction [3], and some argue that addiction to SNSs has grown since the latest technologies (tablets, smartphones) arrived on the scene [2]. It is still difficult to find reliable statistics concerning the prevalence of SNS addiction, however [20]. Studies on addiction to Facebook have focused mainly on samples of undergraduate students, reporting prevalence rates ranging from 1.6 to 21.7% [1]. Whether it is actually addictive or not, social networking excessively and compulsively is unlikely to have positive effects over time, and can be defined, quite simply, as unhealthy [2, 21]. In fact, the outcome of some research points to SNS addicts experiencing problems in the sphere of their emotions and social relations, and with their physical health and performance [42]. A disordered SNS use also seems to lead to a heightened susceptibility to substance and other addictions in undergraduate students [26]. In older adolescent populations, several studies have found associations between inappropriate substance use and a behavioral dependence apparently associated with spending too much time on Facebook [29], and a problematic Internet usage. For instance, when [31] examined Internet addiction and the factors associated with it in 1392 teenagers (13 to 18 years old), the use of alcohol emerged as a risk factor for a diagnosis of addiction to the Internet. This implies that substance use may be associated with problematic social networking sites usage (PSNSU). Recent research findings [50] also point to adolescents becoming addicted to Internet and experimenting with substance use having family-related issues in common. These may involve a more conflictual relationship with their parents, brothers and/or sisters who routinely drink alcohol, the impression that parents do not disapprove of adolescents drinking or smoking, and dysfunctional families generally. It seems reasonable to expect factors relating to the family domain that have anything to do with illegal substance use (such as a teenage sibling who drinks alcohol) to relate to PSNSU as well. Along much the same lines, the association between the experience of gambling and substance use (as demonstrated in earlier research by [15]), may be linked with PSNSU too.

The present study focused on seeking a link between PSNSU and substance use by young adolescents.

## Methods

### Material and participants

A survey called “Pinocchio” was implemented in the academic year 2013–2014 at several secondary schools in the city of Padua (north-east Italy), which has a population of young adolescents numbering around 8000. The study sample included 1325 pupils in years 6 to 8 (11- to 13-year-olds) at 8 different schools. To obtain a sample with an equal distribution in the area, one or two secondary schools from each of the 6 school districts in the city were recruited from among those volunteering to participate in a program conducted at their schools that focused on the prevention of underage drinking and smoking. The pupils anonymously answered a self-administered, ad hoc questionnaire that was developed in the light of a previous study by [17], and presented to participants by a team managing the prevention program. Only pupils with objective difficulties (due to mental disability or a poor knowledge of the Italian language) for the purposes of understanding and answering the questionnaire were excluded.

For all the pupils enrolled, the parents were asked to give their written informed consent to their children’s participation in the survey. The pupils’ verbal assent was also required before they started to complete the baseline questionnaire. One hundred and six parents withheld their consent and their children were excluded. None of the pupils refused to take part in the study.

The questionnaire contained 106 multiple-choice items and touched on all the factors known to have a potential association with risks to behavioral health, i.e. social sphere and demographics, family setting, peers, personality, behavioral factors [8].

The variables measured for each domain entering the model as covariates are shown in Table 1; some variables were categorized as a dummy variables (shown in the same table). The “Gambling” variable was derived from answers of the section “Behavioral domain”, to explicit questions referring to video poker, online betting, or scratch-and-win cards, as shown in Table 1. The substance use variables, shown in Table 2, are used to derive a latent factor measuring recent substance use, the values of the correlations between the variables are shown in Table 3.

**Table 1:** The questionnaire administered, divided into domains associated with health risk factors

Domain risk factors	Variable name	Domain
Socio-demographic factors	Age	*How old are you?*
	Sex	*Are you (male or female)?*
	Nationality	*What’s your nationality?*
Family setting	Separated parents	*Do your parents live together?* (Yes/No)
	Father’s alcohol consumption	*Does your father drink alcohol?* (Never/Rarely/Once a month/ Once a week/ Every day), after dichotomized as (Yes/No)
	Father’s smoking	*Does your father smoke?* (Yes/No)
	Mother’s alcohol consumption	*Does your mother drink alcohol?* (Never/Rarely/Once a month/ Once a week/ Every day) after dichotomized as (Yes/No)
	Mother’s smoking	*Does your mother smoke?* (Yes/No)
	Sibling’s alcohol consumption	*Does your sibling drink alcohol? (Never/Rarely/Once a month/ Once a week/ Every day) after dichotomized as (Yes/No)*
	Sibling’s smoking	*Does your sibling smoke?* (Yes/No)
	Education	*How would you define the education that you have received from your parents as regards obeying rules?* (Flexible/Rigid/No rules)
	Rules for returning home	*When you go out with friends, are you asked to be back by a certain time?* (Yes/No/I never go out with friends)
	Weekly pocket money	*Do you have weekly pocket money?* (Yes/No)
Peer domain	Size of group of friends	*How large is your group of friends?* (No fixed group/2–4 friends/5–9 friends/10–20 friends/> 20 friends)
	Decision-maker in group of friends	*Who makes decisions in your group of friends?* (“I usually decide what we do”/“We decide together”/ “Others decide for me”)
	Friends’ alcohol consumption	*Do your friends drink alcohol?* (Never/Rarely/Once a month/ Once a week/ Every day)
	Friends’ smoking	*Do your friends smoke*? (Yes/No)
Personality domain	Parish groups/Volunteering/ Scouting	*Do you often go to parish/ volunteering/ scouting groups?* (Yes/No)
	Artistic activities	*Do you engage in artistic activities?* (Yes/No)
	Playing sports	*Do you play sports?* (Yes/No)
	Playing competitive sport	*Do you play competitive sports?* (Yes/No)
	Obeying rules	*When people ask you to respect the rules:* (“Always obeys the rules/No respect for rules”)
	Average school mark	*What are your average school marks across subjects?* (4 or less/5/6/7/8/9/10)
Behavioral domain	Hours of sleep	*How many hours do you sleep at night?* (5/6/7/8/9/10/More than 10)
	Time of returning home in the evenings	*What time do you return home in the evening?* (18.00/19.00/20.00/21.00/22.00/23.00/24/00/After midnight) after dichotomized as From 18 to 21:59 h / After 22:00 h
	Text messages sent	*How many text messages do you send a day?*(Number of text messages). Categorized as: 0–15 / 16–99 / 100–499 / ≥500
	Hours spent playing with videogames	*On average, how many hours a day do you play with videogames?* (Number of hours)
	Hours spent watching TV	*On average, how many hours a day do you watch television?* (Number of hours)
	Scratch-and-win cards	*Have you ever bought scratch and win cards?* (Yes/No)
	Video poker	*Have you ever bet money at video poker?* (Yes/No)
	Online betting	*Have you ever placed bets on the internet?* (Yes/No)

**Table 2:** Definition of the variables Substance use and Problematic social networking site usage (PSNSU)

Variable	Question
Substance use	*Have you drunk an alcoholic beverage at least once in the last month?* (Yes/No)
	*Have you drunk an energy drink at least once in the last month?* (Yes/No)
	*Have you smoked at least once in the last month?* (Yes/No)
PSNSU	*Do you ever stay up late and get up early in order to spend more time on social networking sites (Facebook, Netlog, Twitter, ...)?* Never/Rarely/Sometimes/Often/Always)
	*Do you feel anxious if you cannot connect to the social network for a while?* (Never/Rarely/Sometimes/Often/Always)
	*Have you ever spent more time on social networking sites than you had intended?* (Never/Rarely/Sometimes/Often/Always)
	*Have you ever neglected homework, sports activities, time with friends, and so on, in order to spend more time on social networks?* (Never/Rarely/Sometimes/Often/Always)
	*How often do you try to cut down the amount of time you spend on social networks and fail?* (Never/Rarely/Sometimes/Often/Always)
	*Have you ever thought, ‘I’ll carry on just for a few more minutes’ when on social networks?* (Never/Rarely/Sometimes/Often/Always)

**Table 3:** Correlations between variables and rotated factor loadings in the factor analysis

	Correlation			Factor 1
	*Smoking*	*Energy drinks*	*Alcohol*	
*Smoking*	1,00			0.78
*Energy drinks*	0,33	1,00		0.72
*Alcohol*	0,29	0,28	1,00	0.67

The severity of any PSNSU was ascertained from the pupils’ scores on 6 self-rated items, based on those used by Guzzo et al. [22] to investigate social network addiction disorder. More specifically, the questions listed in Table 2 each refer to one of six criteria of substance dependence as established by DSM-IV [18] (tolerance, withdrawal, use of increasing amounts, repeated attempts to quit, activities given up in order to use, too much time spent on use, physical problem related to use).

Answers were given by means of a Likert scale (where 0 meant *never*, 1 meant *rarely*, 2 *sometimes*, 3 *often*, and 4 *always*). It was assumed that a pupil who reported a diagnostic criterion at least sometimes was affected by the corresponding symptom: since the DSM-IV declares that meeting 3 or more diagnostic criteria can be considered dependence, we dichotomized the variable PSNSU as having at least 3 such symptoms. We tested the scale’s reliability coefficient using Cronbach’s alpha: it amounted to 0.76, which could be considered “acceptable”. This measure of PSNSU was adopted because there was no validated tool available for use with young Italian adolescents at the time of our study.

### Statistical methods

First, we calculated proportions and 95% confidence intervals of PSNS users.

Then a preliminary bivariate analysis was run to identify any variables that might be confounders. In particular, the χ^2^ test was applied to test the difference in how the categorical variables were distributed by PSNS usage, while Student’s t-test was used to check for differences in the means of the continuous variables, again by PSNS usage.

A factor analysis was conducted using variables related to substance use, that is smoking, or energy drink or alcohol consumption, for measuring recent substance consumption. We, also, analyzed a polychoric correlation matrix to verify correlations between binary variables concerning smoking, or energy drink or alcohol consumption during the previous month.

The factor analysis revealed that only one factor had an eigenvalue larger than 1 (the eigenvalues were 1.57, − 0.10, and − 0.18 for factors 1, 2, and 3, respectively). The rotated factor loadings with Factor 1 for the different types of substance use are given in Table 3. The likelihood ratio test of independence against the saturated model had a *p* for the χ^2^ test = < 0.001. To see whether it was appropriate to considering only one factor, we ran a confirmatory factor analysis. By constraining to 1 the parameter related to smoking, we obtained satisfactory goodness of fit indexes: the *p*-value related to the chi-square test was 0.15, so we can accept the hypothesis of a good fit. Remarkably good values were also obtained for the Tucker-Lewis and the Comparative Fit indexes, which were 0.96 and 0.97, respectively. The adjusted (0.92) and unadjusted (0.98) goodness of fit indexes were satisfactory too. The eight scores for the Factor 1 latent variable, measuring recent substance use, were then collapsed into three categories: no use (pupils who reported no consumption in the last month); medium-level use (pupils who used only one of the three substances in the last month); and high-level use (different combinations of use of more than one substance).

Finally, an ordered logistic regression was performed: the latent factor measuring recent substance use, categorized into three level, was entered as dependent variable, problematic SNS usage was considered as the independent variable, and the potential confounding factors as covariates. We also used the option to test the proportional odds (or parallel lines) assumptions for each variable, and we constrained the variables that met these assumptions.

The STATA software, ver. 12, was used for all the statistical analyses.

## Results

Slightly more than half (51.4%) of the 1325 pupils enrolled were boys, and most of the pupils (76.5%) were Italian. The sample was a mean 12.4 years old (with a SD of 0.97 years).

**Fig. 1:**
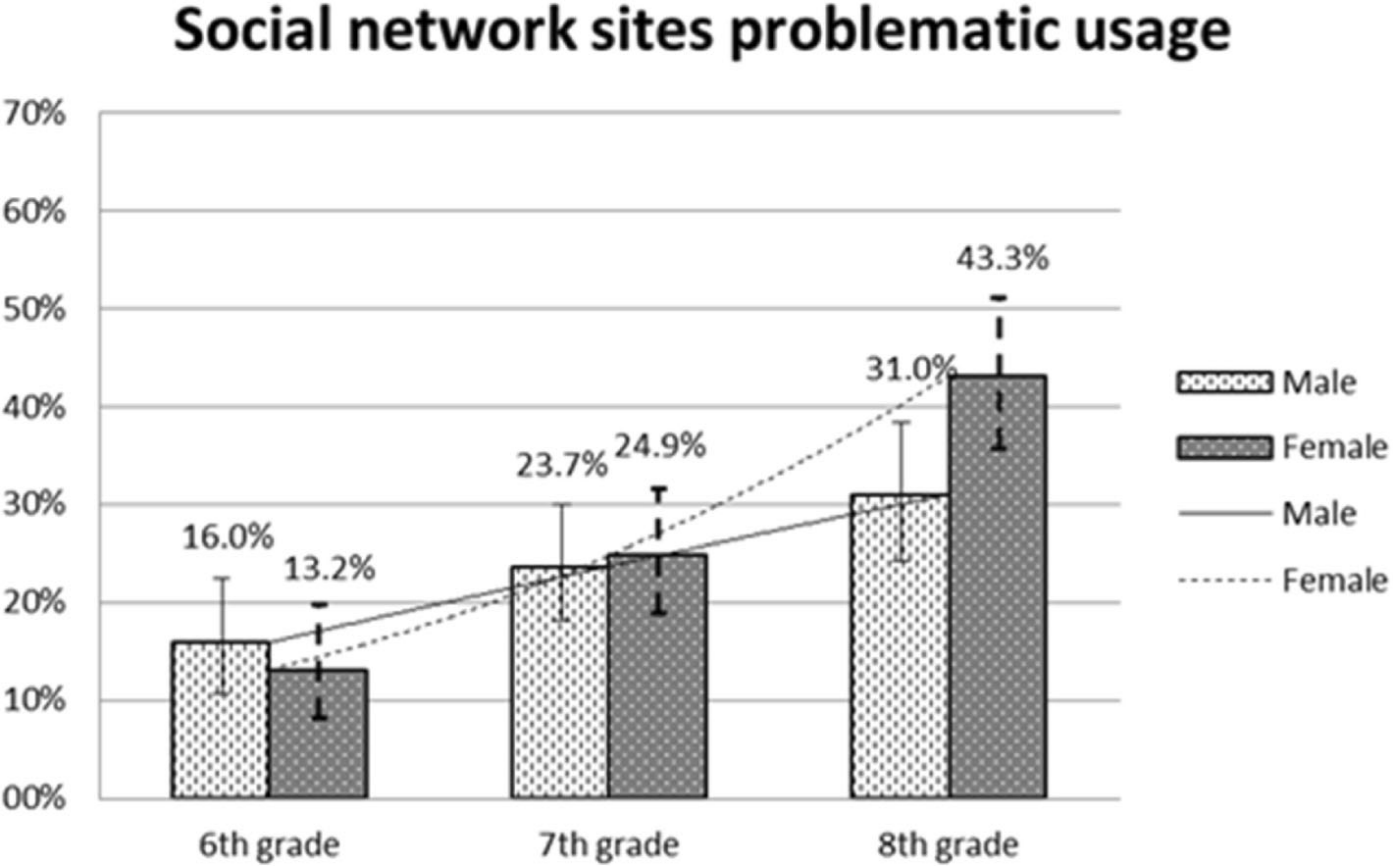
Proportion (%) of adolescents classified as problematic social networking site users (beyond the 75th percentile) by sex and school grade

Figure 1 shows, for each school year, the percentage of pupils classifiable as problematic SNS users. This percentage increased with age (13.2% in year 6, 24.9% in year 7, and 43.3% in year 8), and girls were more affected than boys (with 16.0%, 23.7%, and 31.0% in years 6, 7 and 8, respectively).

The adolescents who had smoked in the previous month amounted to 7.4% (95%CI 6.0–8.9%) of the sample, while 14.7% (95%CI 12.9–17.0) had consumed energy drinks, and 18.3% (95%CI 16.2–20.6%) had drunk alcoholic beverages. An overall 72.2% of the pupils had used none of these substances in the previous month, while 18.6% had used one, 6.7% had used two, and 2.8% had used all three.

The results concerning the association between PSNSU and our study population’s socio-demographic, family, personality, and behavioral characteristics are shown in Tables 4 and 5. Among the variables tested in the bivariate analysis, almost half of the young adolescents who sent 500 or more text messages a day, reported problematic use of SNS (47.5%), amounting to about 18% more cases of self-reported PSNSU than among the individuals who sent 100–499 text messages a day (29.0%), about 30% more than those who sent 16–99 (19.3%), and just over 40% more than those who sent up to 15 text messages a day (7.1%). The proportion of cases of PSNSU among the adolescents who reported having bought scratch cards, or wagered money on video poker games, or placed bets on the Internet (26.7% of the sample) was more than 10% higher than among the respondents not reporting any gambling behavior (15.2% of the sample). The percentages of respondents with self-reported PSNSU were always higher among those who had reportedly smoked or consumed alcohol or energy drinks in the previous month than among those who had not, i.e.: 52.2% vs 16.5% for smoking; 35.4% vs 15.4% for alcohol; and 39.4% vs 15.5% for energy drinks. Table 6 shows the results of the ordered logistic regression. PSNSU was associated with a latent factor called ‘recent substance use’ in a fully-adjusted model (*p* < 0.05). In other words, problematic SNS usage (OR 2.93; 95% CI 1.77–4.85), having siblings (OR 4.81; 95% CI 1.60–14.42), sending at least 500 text messages a day (OR 1.34; 95% CI 1.02–1.76), and having experience of betting in internet, playing video poker or buying scratch cards (OR 2.47; 95% CI 1.53–3.96) increased the likelihood of being a substance user with proportional odds, whereas attending parish group (OR 0.57; 95% CI 0.35–0.93) lowered the likelihood of this happening.

**Table 4:** Association of the socio-demographic, family domain, peer domain, personality domain, and behavioral domain factors with problematic social networking site usage (PSNSU)

VARIABLES		MODALITIES	PSNSU Yes (*n* = 241)	*p*
SOCIO-DEMOGRAPHIC	*Sex*	Male (*n* = 653)	17.3%	0.085
		Female (*n* = 591)	21.2%	
	*Nationality*	Italian (*n* = 969)	18.3%	0.002
		Not Italian (*n* = 204)	27.9%	
FAMILY DOMAIN	*Father’s alcohol consumption*	Yes (*n* = 363)	25.3%	< 0.001
		No (*n* = 842)	16.3%	
	*Mother’s alcohol consumption*	Yes (*n* = 176)	25.0%	0.038
		No (*n* = 1031)	18.3%	
	*Sibling’s alcohol consumption*	Yes (*n* = 42)	38.1%	0.001
		No (*n* = 1005)	18.0%	
	*Father’s smoking*	Yes (*n* = 291)	26.1%	< 0.001
		No (*n* = 964)	16.8%	
	*Mother’s smoking*	Yes (*n* = 183)	23.5%	0.093
		No (*n* = 1075)	18.2%	
	*Sibling’s smoking*	Yes (*n* = 88)	33.0%	0.001
		No (*n* = 1012)	18.0%	
	*Weekly pocket money*	1–10 €/week (*n* = 250)	17.6%	0.023
		More than 10 €/week (*n* = 120)	28.3%	
		No (*n* = 894)	18.1%	
PEER DOMAIN	*Friends’ smoking*	Yes (*n* = 352)	35.5%	< 0.001
		No (*n* = 896)	12.6%	
	*Friends’ alcohol consumption*	Yes (*n* = 99)	49.5%	< 0.001
		No (*n* = 1034)	16.5%	
PERSONALITY DOMAIN	*Obeying rules*	Always obeys the rules (*n* = 886)	14.5%	< 0.001
		No respect for rules (*n* = 360)	30.8%	
	*Parish groups*	Yes (*n* = 752)	16.1%	0.002
		No (*n* = 507)	23.1%	
	*Volunteering*	Yes (*n* = 174)	19.9%	0.086
		No (*n* = 1081)	14.4%	
	*Artistic activities*	Yes (*n* = 403)	15.4%	0.024
		No (*n* = 853)	20.8%	
	*Playing sport*	Yes (*n* = 1085)	17.7%	0.002
		No (*n* = 177)	27.7%	
BEHAVIORAL DOMAIN	*Returning home in the evenings*	From 18 to 21:59 h (*n* = 665)	21.2%	0.028
		After 22 h (*n* = 168)	29.2%	
	*Hours of sleep*	> 8 h (*n* = 1066)	15.9%	< 0.001
		< 8 h (*n* = 195)	36.9%	
	*Text messages sent*	0–15 text messages (*n* = 297)	7.1%	< 0.001
		16–99 text messages (*n* = 415)	19.3%	
		100–499 text messages (*n* = 314)	29.0%	
		≥500 text messages (*n* = 80)	47.5%	
	*Gambling*	Yes (*n* = 419)	26.7%	< 0.001
		No (*n* = 845)	15.2%	
	*Hours spent watching TV*	From 30 min to 2 h / day (*n* = 721)	18.5%	0.009
		More than 2 h / day (*n* = 333)	23.7%	
	*Smoking in the last month*	Yes (*n* = 90)	52.2%	< 0.001
		No (*n* = 1175)	16.5%	
	*Alcohol consumption in the last month*	Yes (*n* = 229)	35.4%	< 0.001
		No (*n* = 1036)	15.4%	
	*Energy drinks consumption in the last month*	Yes (*n* = 184)	39.7%	< 0.001
		No (*n* = 1081)	15.5%	

The following variables were tested and found unassociated (*p* > 0.10) with PSNSU (data not shown): Separated parents, Rules for returning home, Older sibling > 13 years old, Size of group of friends, Decision-maker in group of friends, Hours spent playing with videogames, Education

**Table 5:** Means and standard deviations of variables by problematic social networking site usage (PSNSU) group

VARIABLE	PSNSU Yes(*n* = 276) Mean ± SD	PSNSU No (*n* = 796) Mean ± SD	*p*
Age (yr)	12.62 ± 0.99	12.17 ± 0.98	< 0.001
Average school marks	6.97 ± 1.02	7.36 ± 0.98	< 0.001

**Table 6:** Ordered logistic regressions analysis of associations between recent substance use, derived from the latent variable, and problematic social networking site usage group: odds ratio and 95% confidence intervals (CI), *p* value

Recent substance (alcohol, cigarettes and energy drinks) use		Odds ratio	[95% CI]	*p*
Significant Proportional Odds				
Parish Group [ref. No]		0.57	0.35–0.93	0.025
≥500 text messages sent [ref. 0–15]		1.34	1.02–1.76	0.038
Gambling Yes [ref. No]		2.47	1.53–3.96	0.000
Siblings’ alcohol consumption Yes [ref. No]		4.81	1.60–14.42	0.005
Problematic social networking site usage Yes [ref. No]		2.93	1.77–4.85	0.000
Significant Not Proportional Odds				
No vs Middle-High	Male	0.75	0.45–1.24	0.256
	No obeying rules	1.38	0.82–2.30	0.224
	Hours of sleep < 8 h	1.61	0.89–2.93	0.117
	Weekly pocket money > 10 Euro	0.92	0.63–1.33	0.644
	Siblings’ smoking Yes	0.77	0.35–1.69	0.522
No-Middle vs High	Male	0.34	0.15–0.76	0.009
	No obeying rules	5.75	2.66–12.40	0.000
	Hours of sleep < 8 h	3.34	1.50–7.41	0.003
	Weekly pocket money > 10 Euro	2.06	1.25–3.39	0.005
	Siblings’ smoking Yes	3.05	1.19–7.76	0.020

*CI* confidence interval

The following variables were tested and found unassociated (*p* < 0.05) with recent substance use (data not shown): Age, Nationality, Artistic activities, Playing sport, Returning home in the evenings, Hours spent watching TV, Father’s smoking, Friends’ smoking, Father’s alcohol consumption, Friends’ alcohol consumption, Mother’s alcohol consumption and Average school marks

## Discussion

This study demonstrates that young adolescents who are problematic SNS users are more likely to have recent experience of drinking alcoholic beverages, smoking or using energy drinks, after adjusting for socio-demographic factors, family, peers, personality, and behavioral risk factors that have already revealed a link with teenage substance use [22].

Research addressing this topic has been virtually non-existent to date [30], but our results are in line with the one other report [26] on PSNSU and substance misuse, which was a cross-sectional analysis conducted on university undergraduates. In their case, an excessive social networking site usage was positively associated with emotion regulating problems and drinking issues. The Authors concluded that a disordered SNS usage seems to be one of the signs of difficulties with regulating emotions and a higher likelihood of substance abuse and addictions.

A possible explanation for the relationship observed in our study between PSNSU and substance consumption by adolescents can be sought in the amount of peer influence involved in social networking. Peer influence plays an important part in adolescence, as this is a time of life when individuals develop a new identity, make new friends, and join different peer groups. Meanwhile, their parents’ influence tends to decrease [43]. Any adolescent’s peers can be strongly influential, and friends may encourage each other to experiment with substances and take risks, exerting a normative pressure to do so [46]. Plenty of evidence has emerged of how a given adolescent’s use of tobacco and alcohol is often linked to the use of these substances by their friends [6, 24]. Our data seem to point in this direction too, highlighting the association between having friends who drink alcohol and having smoked or consumed alcohol or energy drinks in the previous month. In addition, the analysis shows the relationship between having friends who drink alcohol and PSNSU, which could indicate that online exchanges with friends might mediate peer influence processes (as regards adolescent cigarette and alcohol use) by conveying information about peers’ risk-taking behavior. [27] found that smoking and drinking by a sample of 10th-grade American students were significantly associated with having friends who posted pictures of partying or drinking online. The Authors concluded that an adolescent’s risk-taking behavior was directly affected by exposure to online content, and significantly correlated with their friends’ risk-taking behavior.

Online media often contain references to smoking and drinking, including descriptions and photographs of young people’s drinking experiences [37]. Judging from a review of MySpace profiles, adolescents often boast of being familiar with adult-oriented behavior [23], such as smoking and alcohol drinking. When adolescents create and display social network content, this may be seen by their peers as a model to imitate, and social networks have the potential to strongly influence an adolescent’s alcohol and tobacco use [13]. Applying social learning theories [4, 19, 41] to modern media suggest that adolescents who see others drinking or smoking, and apparently suffering no unpleasant effects of their behavior, will be more inclined to follow suit. We also know from social learning theory that messages conveyed by the media concerning people’s motives for certain behavior, and its pleasant associations and positive outcomes, are bound to have their appeal [41]. In another vein, the association between social networks and substance consumption can also be explained by the influence of marketing on the young. A social media case study [48] on a number of brands of alcoholic beverages found them abundantly present online, in content generated by marketers and users. The study described Facebook profiles in which advertisers and customers commented on these beverages, as well as competitions, videos, recipes, apps and games inciting viewers to engage with the marketers’ content. For instance, Mart et al. counted more than 50,000 Facebook groups that had to do with alcohol in some way - over and above the alcohol manufacturers’ direct marketing material [34]. There are promotions and events on the Facebook platform that relate to alcohol brands [Freeman B, Chapman S], and – despite the World Health Organization’s ban on all forms of tobacco advertising, promotion and sponsorship (in its Framework Convention on Tobacco Control), a study that checked for any such promotional activities by two British-American global tobacco brands on Facebook found more than 500 items across a variety of Facebook subsections [14].

On the other hand, the results of our study indicate that the affiliation to parish groups reduces the probability of adolescents experimenting with illicit substances. In our sample, the proportion of respondents with PSNSU was also lower among those who reported having a religious faith than among those who considered themselves atheists. These findings seem to suggest that, in early adolescence at least, religion has a part to play in helping children to mature and protecting them against risk-taking behavior [10]. In spite of the paucity of literature currently available on the topic, several reports have described how adolescents’ religiousness relates to their risk-taking behavior. For instance, [32] found that religious affiliation helps to protect against delinquency, and [5] found a role for religion in preventing adolescent drug use. Other researchers noted that religious adolescents were less likely to abuse of marijuana or steroids, or to drive under the influence of alcohol [7, 47, 49].

Overall, our data bring to light an alarming picture, considering that most of the adolescents enrolled in our sample were not old enough to access Facebook, for instance, which establishes that members should be at least 13 years old [12]. Even greater cause for concern emerges from an Israeli study on how the parents of 195 Facebook users aged between 8 and 17 supervised their offspring’s Internet usage. The Authors reported that these parents were less inclined to monitor their children’s activities on Facebook than parents of older teenagers [9]. The researchers suggested that this was due to parents assuming that younger adolescents’ online behavior would be more innocuous (playing games, chatting to friends) than might be the case of older teenagers (13+). If this attitude is shared by the parents of most underage Facebook users, young adolescents would be more at risk than older teenagers (as the latter would be supervised by their parents) [25]. Our study confirmed the link between PSNSU and risk-taking behavior such as substance use at a very young age, highlighting the importance of SNS usage being included in schemes designed to prevent substance abuse and other risk-related behavior in early adolescence. A previous study [16, 18] had also shown that parents who supervise their children’s media usage have the effect of safeguarding their academic, social, and physical development. Pediatricians and general physicians are in a good position to give families scientifically sound advice, and to urge parents to monitor their children’s time on the Internet with care, as this can have far-reaching effects on their health.

When it comes to interpreting the results of the present research, a number of limitations need to be considered. For a start, this was a cross-sectional study, which makes it difficult to infer causality, especially as regards the one- or two-way direction of the association between substance use and PSNSU. Another weakness lies in that substance abuse is always a sensitive matter for adolescents, and our findings may be biased by their having exaggerated or played down their own behavior. We can assume, however, that this potential source of bias was contained by our use of a self-administered anonymous questionnaire. A third limitation concerns our requesting that respondents mention *any* alcohol drinking, cigarette smoking, or energy drink use in the previous month, so we also captured experimental sipping and puffing as well as more regular consumption patterns. This approach was used because, given the young age of our sample, any use at all (even in small quantities) is important: it can be seen as a challenge that draws young adolescents towards further risk behavior. That is why we preferred to adopt the type of question formulated by authors such as Peterson et al. [40], and to ask participants if they had drunk or smoked at all during the previous month. Other authors had also found it more useful to ask if respondents had ever engaged in a given undesirable behavior in the past, rather than whether they were doing so in the present, because past events are less threatening [44].

Be that as it may, our questions did not distinguish between substance use with and without parents’ permission (such as a sip of wine for a toast at a birthday party), and it will be necessary to consider this issue in further studies [28].

## Conclusion

In conclusion, this study revealed an association between PSNSU and other behavioral problems in young adolescents. Health promotion schemes that aim to intervene on several behavioral fronts should include the issue of PSNSU in this age group. When cases of an unhealthy use of social networking sites are identified, it is important to bear in mind that the approach to treating adolescent PSNSU should never involve total abstinence. Using the Internet has become an essential part of an adolescent’s schooling and recreational culture. Efforts should focus on ensuring that their use of this medium (and especially of social networking sites) is kept under control. Relapsing PSNSU can be prevented by means of strategies developed in the setting of cognitive-behavioral therapies [11], such as those well described by [51]. These include, for example: (a) hindering adolescents’ excessive Internet use by identifying their usage patterns and then disrupting them by rescheduling their spare time; (b) using external interferences in the form of events and activities that induce them to log off; (c) setting limits for the amount of time they are allowed to spend in Internet; (d) preventing them from accessing a particular application (beyond their control); (f) drawing up a list of all the things a given adolescent used to do before becoming too attached to Internet, such as sports, or hobby group activities.

## Abbreviations

PSNSU: Problematic social networking site usage
